# Effect of a Smartphone App (S-Check) on Actual and Intended Help-Seeking and Motivation to Change Methamphetamine Use Among Adult Consumers of Methamphetamine in Australia: Randomized Waitlist-Controlled Trial

**DOI:** 10.2196/55663

**Published:** 2024-07-03

**Authors:** Krista J Siefried, Florence Bascombe, Brendan Clifford, Zhixin Liu, Peter Middleton, Frances Kay-Lambkin, Jack Freestone, Daniel Herman, Michael Millard, Maureen Steele, Liam Acheson, Carl Moller, Nicky Bath, Nadine Ezard

**Affiliations:** 1 The National Centre for Clinical Research on Emerging Drugs University of New South Wales Randwick Australia; 2 St Vincent’s Hospital Alcohol and Drug Service Sydney Australia; 3 The National Drug and Alcohol Research Centre, The University of New South Wales Sydney Australia; 4 Institute for Global Health University College London London United Kingdom; 5 Central and North-West London NHS Foundation Trust London United Kingdom; 6 University College London Hospitals NHS Foundation Trust London United Kingdom; 7 New South Wales Drug and Alcohol Clinical Research and Improvement Network New South Wales Ministry of Health Sydney Australia; 8 Healthdirect Australia Sydney Australia; 9 School of Medicine and Public Health College of Health, Medicine and Wellbeing University of Newcastle Newcastle Australia; 10 ACON Sydney Australia; 11 The Kirby Institute University of New South Wales Sydney Australia; 12 The Practice Healthcare Sydney Australia; 13 Clinical Research Unit for Anxiety and Depression St Vincent's Hospital Sydney Australia; 14 LGBTIQ+ Health Australia Sydney Australia

**Keywords:** methamphetamine, smartphone app, behavior change, help-seeking, motivation to change, mHealth, mobile health, app, apps, application, applications, smartphone, smartphones, motivation, motivational, RCT, randomized, controlled trial, controlled trials, drug, drugs, substance use, engagement, substance abuse, mobile phone

## Abstract

**Background:**

Interventions are required that address delays in treatment-seeking and low treatment coverage among people consuming methamphetamine.

**Objective:**

We aim to determine whether a self-administered smartphone-based intervention, the “S-Check app” can increase help-seeking and motivation to change methamphetamine use, and determine factors associated with app engagement.

**Methods:**

This study is a randomized, 28-day waitlist-controlled trial. Consenting adults residing in Australia who reported using methamphetamine at least once in the last month were eligible to download the app for free from Android or iOS app stores. Those randomized to the intervention group had immediate access to the S-Check app, the control group was wait-listed for 28 days before gaining access, and then all had access until day 56. Actual help-seeking and intention to seek help were assessed by the modified Actual Help Seeking Questionnaire (mAHSQ), modified General Help Seeking Questionnaire, and motivation to change methamphetamine use by the modified readiness ruler. *χ*^2^ comparisons of the proportion of positive responses to the mAHSQ, modified General Help Seeking Questionnaire, and modified readiness ruler were conducted between the 2 groups. Logistic regression models compared the odds of actual help-seeking, intention to seek help, and motivation to change at day 28 between the 2 groups. Secondary outcomes were the most commonly accessed features of the app, methamphetamine use, feasibility and acceptability of the app, and associations between S-Check app engagement and participant demographic and methamphetamine use characteristics.

**Results:**

In total, 560 participants downloaded the app; 259 (46.3%) completed eConsent and baseline; and 84 (32.4%) provided data on day 28. Participants in the immediate access group were more likely to seek professional help (mAHSQ) at day 28 than those in the control group (n=15, 45.5% vs n=12, 23.5%; *χ*^2^_1_=4.42, *P*=.04). There was no significant difference in the odds of actual help-seeking, intention to seek help, or motivation to change methamphetamine use between the 2 groups on the primary logistic regression analyses, while in the ancillary analyses, the imputed data set showed a significant difference in the odds of seeking professional help between participants in the immediate access group compared to the waitlist control group (adjusted odds ratio 2.64, 95% CI 1.19-5.83, *P*=.02). For participants not seeking help at baseline, each minute in the app increased the likelihood of seeking professional help by day 28 by 8% (ratio 1.08, 95% CI 1.02-1.22, *P*=.04). Among the intervention group, a 10-minute increase in app engagement time was associated with a decrease in days of methamphetamine use by 0.4 days (regression coefficient [β] –0.04, *P*=.02).

**Conclusions:**

The S-Check app is a feasible low-resource self-administered intervention for adults in Australia who consume methamphetamine. Study attrition was high and, while common in mobile health interventions, warrants larger studies of the S-Check app.

**Trial Registration:**

Australian New Zealand Clinical Trials Registry ACTRN12619000534189; https://www.anzctr.org.au/Trial/Registration/TrialReview.aspx?id=377288&isReview=true

## Introduction

Frequent use of methamphetamines is associated with adverse physical and mental health consequences [1,2], and Australia reports one of the highest rates of methamphetamine use and dependence in the world [2]. Since 2010, associated harms have been steadily increasing [3]. Psychosocial therapies are the primary mode of treatment for methamphetamine use disorder [4], especially as no pharmacotherapy has yet been shown to be effective [5]. Interventions such as cognitive behavioral therapy show modest effects [6], while contingency management has been demonstrated to be effective for outcomes associated both with methamphetamine use and with harm reduction for the duration of the intervention, but lacks widespread uptake [7]. Access to treatment is associated with more positive outcomes [8], but despite this, a gap of up to 10 years has been shown between the onset of problems related to methamphetamine use and presentation to health services for assistance [9].

Delays in access to health services can be due to a perception of “nonproblematic” methamphetamine use among people with methamphetamine use disorder [10,11]. Mental, physical, and social consequences can result from untreated persistent methamphetamine use disorder [9]. The development of a low-threshold intervention to encourage awareness of problems related to methamphetamine use could potentially change behavior and reduce the gaps between use, problems related to use, and treatment access. Health interventions supported by mobile devices (mHealth) are a promising mode of delivering early interventions to people who use drugs. As demonstrated in other mental health and substance use disorders, mHealth offers advantages in privacy and access over traditional face-to-face services and may facilitate help-seeking [12-16]. There are no mHealth interventions such as smartphone apps that have been shown to be effective in encouraging behavioral changes in people who use methamphetamine [17].

The S-Check intervention is targeted toward people who use methamphetamine, who do not necessarily identify problems related to its use [18], and are curious about the relationship between their use and other aspects of their health and well-being. Developed in 2011, the S-Check intervention consists of 4 sessions: a psychosocial assessment with a counsellor (based on Smout et al [19]); a physical health assessment with a medical officer; physical health feedback from a medical officer; and a biopsychosocial feedback, goal setting, and care planning session with a counsellor. The S-Check intervention aims to reduce harms associated with methamphetamine use, support behavior change, and encourage treatment seeking where indicated. When evaluated in 2018 [11], a majority of participants reported that the S-Check intervention was their first contact with general health services for issues related to methamphetamine use. Participants identified harm reduction, including education and advice about safer consumption practices, and the accessible and approachable environment to track their methamphetamine use patterns, as benefits of the model [11].

The S-Check intervention was translated into a smartphone app (“the S-Check app”) that uses the eHealth Behavior Management Model [20] to encourage help-seeking for methamphetamine use. The eHealth Behavior Management Model incorporates the transtheoretical model [20] and the behavioral intent aspect of the theory of planned behavior [21] to create systems that tailor health communication to meet the needs of individual app users through a set of predeveloped messages based on readiness to change. The S-Check app allows app users to assess the risks and harms associated with their methamphetamine use in 5 domains (sexual, lifestyle, physical, psychological, and cognitive health), track their use of methamphetamine and other health measures with an Ecological Momentary Intervention [22], and receive tailored feedback based on persuasive communication principles [20,23,24]. The S-Check app incorporates principles of efficacious behavioral change models [25,26] to reinforce engagement and positive change by encouraging S-Check app users to attend to messages; requesting further inputs from app users; and motivating app users toward change. The S-Check app was piloted with 10 consumers and found to be acceptable and usable by participants [27].

Following the development of the S-Check intervention and the translation to a usable smartphone app, we aimed to assess the effectiveness of the S-Check app to motivate help-seeking among people who use methamphetamine and its effect on motivation to change methamphetamine use. To do this, we undertook a randomized waitlist-controlled trial of the S-Check app.

## Methods

### Trial Design

This study was a digital randomized 28-day waitlist-controlled trial. Participants were allocated 1:1 to one of two study arms: immediate access to the S-Check app (intervention group) or access after a 28-day waitlist period (waitlist control group). The primary end point was day 28, and the primary outcome was between-group differences in help-seeking. Both groups had the option to use the S-Check app through to day 56. Optional qualitative interviews were conducted with consenting participants and will be reported elsewhere. This study is reported in line with the CONSORT (Consolidated Standards of Reporting Trials) guidelines [28], a checklist is available in Table S1 in Multimedia Appendix 1.

### Ethical Considerations

This study was approved by the Human Research Ethics Committee of St Vincent’s Hospital, Sydney, Australia (HREC/18/SVH/196).

### Participants

Inclusion criteria were adults (aged ≥18 years) living in Australia, who had self-reported methamphetamine use at least once in the month before study enrollment and private access to a smartphone (Android or Apple) to day 56 of follow-up. There were no exclusion criteria. Participants were recruited anonymously. This study was promoted through posters and leaflets at community (eg, people who use drugs) and partnering (eg, needle and syringe programs [NSP], medically supervised injecting center, sexual health clinics) organizations, alcohol and other drug service websites, and social media (including location-based dating apps).

### Intervention

The S-Check app was available to download free of charge from Android and iOS app stores. Eligibility screening and provision of the participant information sheet and consent form were completed electronically within the app as an anonymous automated consent procedure [29].

**Figure 1:**
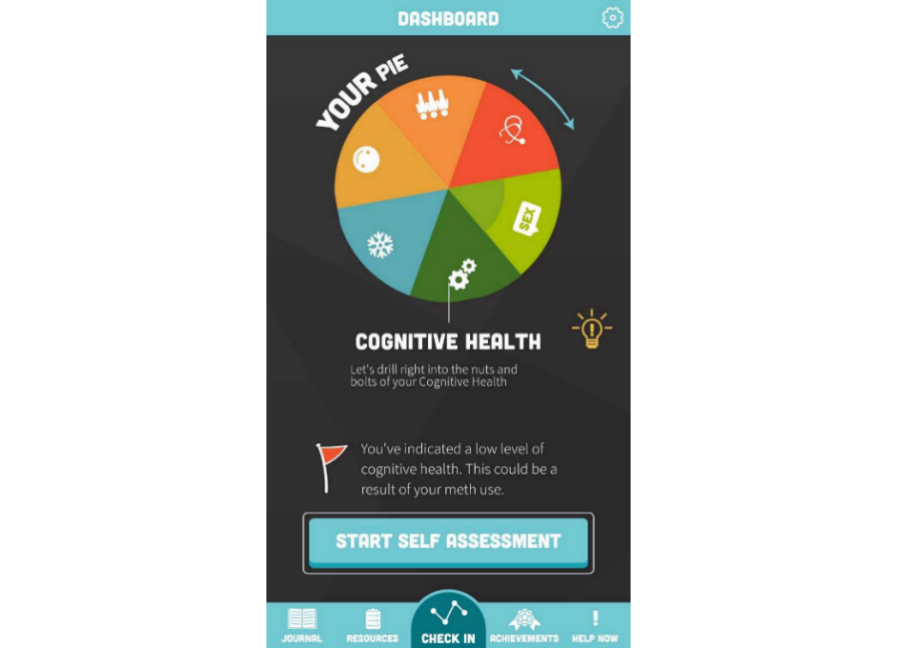
S-Check app dashboard.

**Figure 2:**
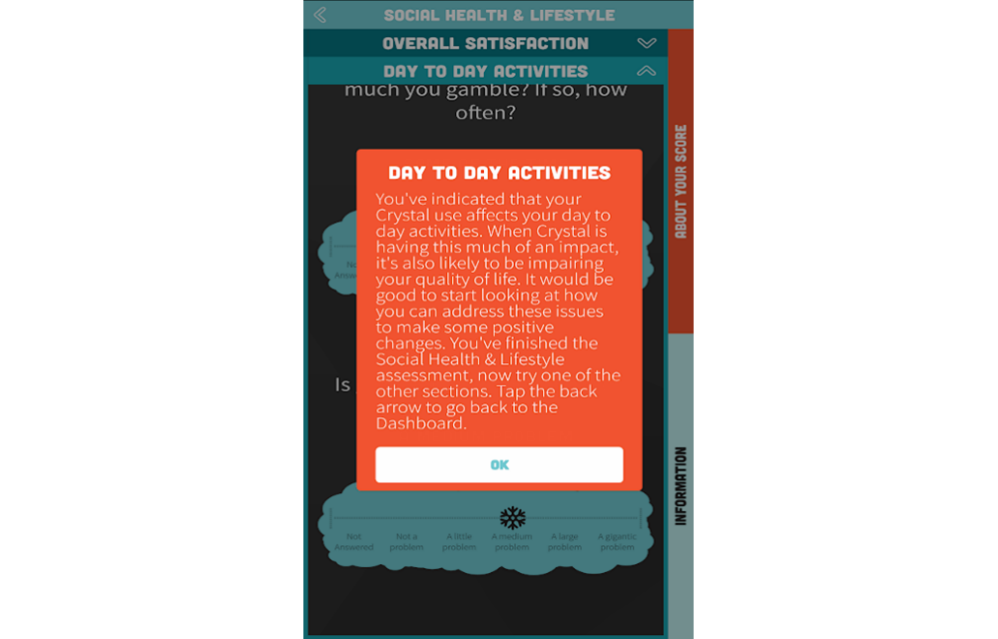
S-Check app generated tailored feedback.

**Figure 3:**
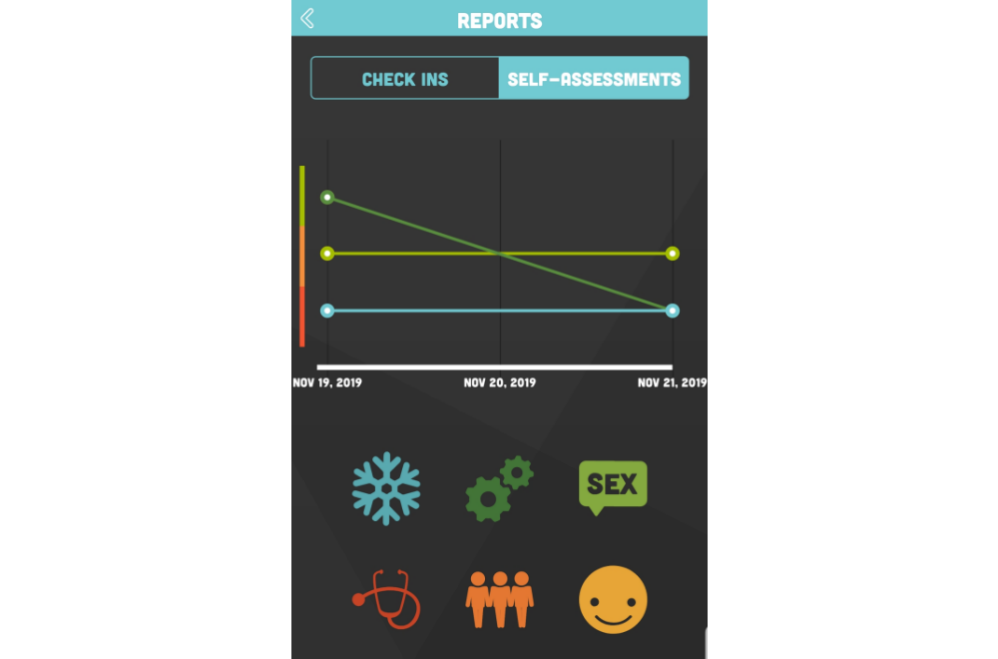
Tracking health over time.

Once accessed, the S-Check app home screen presented a dashboard (Figure 1) to access the S-Check app resources. These included 6 structured self-assessment tools (methamphetamine use, sexual health, social health, psychological well-being, physical health, and cognitive health; Table 1), provision of automated feedback (Figure 2), generation of a referral letter when appropriate to services or further support, relevant up-to-date information and resources about methamphetamine use, and a tool to track methamphetamine use and health impacts of methamphetamine over time (Figure 3)*.*

Data were stored on a secure, encrypted password-protected Customer Relationships Management database integrated into the S-Check app’s unique Application Programming Interface.

**Table 1:** S-Check app self-assessment layers and questionnaires.

Scale		S-Check app layer	Outcome measured	Questions, n
**Self-assessment module: methamphetamine use**				
	Based on ASSIST^a^-Lite List [30]	My use	Frequency of substance use (methamphetamine)	8
	SDS^b^ [31]	How I feel about my use	Methamphetamine dependence	5
**Self-assessment module: psychological well-being**				
	Derived from The Mental State Examination–Mood [32]	My mood rating	Predominant emotion over the past 2 weeks	1
	World Health Organization 5 Well-Being Index Scale [33]	My general well-being	Mental well-being	5
	Kessler Psychological Distress Scale (K10) [34]	Am I feeling ok?	Mental distress	10
	Patient Health Questionnaire [35]	My mental health	Depression	9
	Generalized Anxiety Disorder 7 [36]	Stress and anxiety	Screen for or severity of anxiety	7
**Self-assessment module: social health and lifestyle**				
	Derived from the World Health Organization Quality of Life Scale [37]	Overall satisfaction	Quality of life	3
	Derived from S-Check Biopsychosocial assessment [18]	Day-to-day activities	Impact of use on social factors	10
**Self-assessment module: physical health**				
	Derived from S-Check biopsychosocial and physical health assessment [18]	How you take your [methamphetamine], your medical history, your family’s health, and your body	Effect of drug use on physical health	17
**Self-assessment module: sexual health**				
	Derived from S-Check biopsychosocial assessment [18]	ChemSex, sexual behavior, and sex and drugs	Effect of drug use on sexual health	6
**Self-assessment module: cognitive health**				
	Quality of life in neurological disorders—cognitive function questionnaire [38]	Cognitive health	Cognitive function	8
	A 16-item version of the Prodromal Questionnaire (psychosis screener) [39]	Reality check	Risk of psychosis	16

^a^ASSIST: Alcohol, Smoking and Substance Involvement Screening Test.

^b^SDS: Severity of Dependence Scale.

### Outcomes and Measures

Help-seeking was measured by (1) the modified Actual Help Seeking Questionnaire (mAHSQ; recent help-seeking) [40], (2) the modified General Help Seeking Questionnaire (mGHSQ; intention to seek help) [40], and (3) the modified readiness ruler (mRR; motivation to change behavior). AHSQ and GHSQ are validated to measure intention for and actual help-seeking in mHealth interventions addressing substance use [41] and mental health [42-45]. Both problem-type and the help-source for AHSQ and GHSQ were modified for methamphetamine use [40] over 4 weeks, and for ease of use in the app, response options were collapsed into “professional” and “nonprofessional” help sources. The mAHSQ has 2 binary measures (yes or no) for help-seeking for a problem related to the use of methamphetamine in the past 4 weeks from (1) a professional source (eg, doctor, counsellor, psychologist, specialist, telephone or web-based counseling, etc) and (2) a nonprofessional source (eg, partner, friends, parents, other family members, colleagues, internet) [41,46]. The mGHSQ scale captures intent to seek professional and nonprofessional help in the next 4 weeks, rating each source of help on a 7-point Likert-type scale ranging from 1 (extremely unlikely) to 7 (extremely likely). The mRR is a measure that has demonstrated higher predictive validity than longer measures for readiness to change among people with substance use disorder [47-49]. Participants were asked: “how ready are you to change your use of [nickname of methamphetamine]?” with responses rated and scored on a 10-point Likert-type scale ranging from 1 (not ready) to 10 (trying hard to change). Primary outcomes were measured at baseline (day 0), day 28 (primary end point), and repeated at day 56 for both intervention and control groups.

Secondary outcomes were the most commonly accessed features of the S-Check app (number of times participants accessed each section); number of days of methamphetamine use in the past 28 days (collected at baseline, day 28, and day 56); feasibility and acceptability assessed by the number of participants retained to days 28 and 56 and a qualitative substudy (to be reported elsewhere). Associations were examined between S-Check app engagement over this study’s period (total minutes in-app use) and participant characteristics collected within the S-Check app: age, gender, sexual orientation, education, age of first use of methamphetamine, injection of methamphetamine in the last 4 weeks, treatment history, days of methamphetamine use in the past 28 days, and recent treatment.

For completion of the day 28 and day 56 surveys, participants in both the waitlist and control group were reimbursed with an AUS $25 (approximately US $19 at the time of enrollment) digital grocery voucher, redeemable through the S-Check app.

### Sample Size

We determined the sample size required to evaluate the outcome of actual help-seeking at a power of 0.8 to detect a small to medium effect size (*d*=0.25), and α set at .05. A minimum of 510 participants was required [50] as we anticipated 50% attrition, and 255 participants to remain enrolled to the primary end point (day 28).

### Randomization and Blinding

Randomization was automated within the S-Check app using a JavaScript (Refsnes Data) function whereby a random-access token was generated and participants were allocated to either immediate access to the S-Check app or a waitlist control group. Since participants were automatically enrolled and consented within the app, there was no risk of unblinding to the investigators.

### Statistical Methods

We compared baseline characteristics between the 2 groups using *χ*^2^ for categorical variables, and the *t* test (2-tailed) or Mann-Whitney *U* test for continuous variables.

*χ*^2^ comparisons for the proportion of positive responses to the mAHSQ, mGHSQ (dichotomized to <5 being no intention to seek help and ≥5 being intention to seek help), and mRR (dichotomized to >8 indicating motivation to change) were conducted between the 2 groups at baseline and day 28.

For the mAHSQ, a logistic regression model compared the odds of recent help-seeking at day 28 between the intervention and the control groups, adjusted with the recent help-seeking status at baseline included as a covariate in the model. For the mGHSQ a logistic regression compared the intention of help-seeking score (derived from the mGHSQ dichotomized to <5 being no intention to seek help and ≥5 being intention to seek help) at day 28 between the intervention and control groups, adjusted with the intention to seek help status at baseline included as a covariate in the model. For the mRR, a logistic regression compared the motivation to change behavior (derived from the mRR with a score >8 indicating motivation to change) at day 28 between the 2 groups, adjusted for the corresponding baseline score, by including it as a covariate in the model.

Descriptive statistics were used to report participant access to layers within the app, methamphetamine use, and study retention at days 28 and 56. To analyze the association between demographics and methamphetamine use characteristics and S-Check app use, total engagement time for the intervention group was aggregated from the range of S-Check app use activities (from time of download to day 28). Multiple linear regression was applied to examine which baseline characteristics were predictive of more time engaged with the S-Check app, where engagement time as a dependent variable was log-transformed. The effect of time engaged with the S-Check app on help-seeking (mAHSQ and mGHSQ) and motivation to change (mRR) at day 28 were also examined. Dashboard activity time greater than 60 minutes was treated as outliers, as they were unlikely to accurately reflect true app engagement duration and be imputed with the sample median.

To handle missing data at follow-up, the analysis was conducted initially and primarily based on the retained cohort of observed data, following the recommendation of Jakobson et al [51]. Ancillary analysis based on the full intention-to-treat (ITT) cohort using multiple imputations by chained equations was performed under the missing at-random assumption. Variables in the multiple imputation model included the response to the primary outcome measure at baseline, as well as demographic and methamphetamine use variables in the collected data. The statistical significance level was set at 2-sided .05. Analyses were performed using SAS (SAS Institute) and R (R Foundation for Statistical Computing).

## Results

### Participants

**Figure 4:**
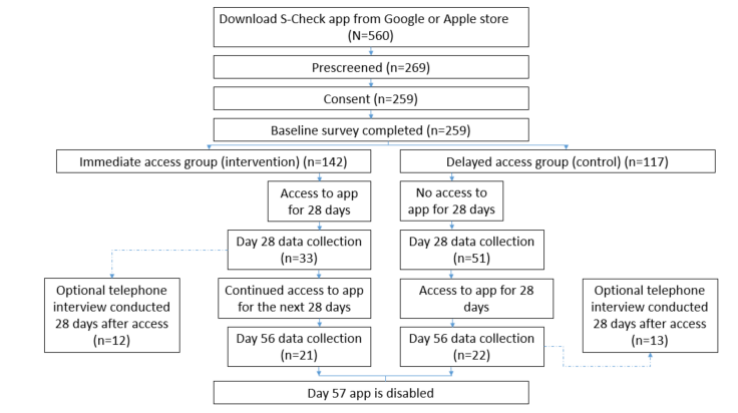
Participant flow diagram.

In total, 560 participants downloaded the app, of which 259 (46.3%) completed the eConsent process and baseline survey. Of these, 84 (32.4%) provided data on day 28. Further, 175 did not provide data at day 28, resulting in a 68% attrition from baseline. A participant flow diagram is presented in Figure 4.

Baseline participant features, summarized by intervention group or control group, are presented in Table 2. There was no significant difference observed between the 2 groups. The majority of the sample (n=159, 61.6%) had not previously accessed treatment services for methamphetamine use, while 55.8% (n=144) had injected methamphetamine in the prior 4 weeks.

**Table 2:** Baseline characteristics of the sample.

Variable			Total eligible participants (n=259)							Participants retained to day 28 (n=84)				
			Control (n=117)		Intervention (n=142)		Total (n=258)^a^		Control (n=50)			Intervention (n=33)		Total, n
Age (years), mean (SD)			38.2 (10.2)		38.9 (11.1)		38.7 (10.7)		39.3 (10.9)			40.7 (10.2)		83
**Sexuality and gender identity, n (%)**														
	Cis gay man	58 (50)		73 (51.4)		131 (50.8)		27 (54)			19 (57.6)		46	
	Cis heterosexual woman	19 (16.4)		20 (14.1)		39 (15.1)		5 (10)			7 (21.2)		12	
	Cis heterosexual man	13 (11.2)		23 (16.2)		36 (13.9)		4 (8)			3 (9.1)		7	
	Nonbinary queer or different	9 (7.8)		10 (7)		19 (7.4)		4 (8)			1 (3)		5	
	Cis bisexual woman	8 (6.9)		7 (4.9)		15 (5.8)		5 (10)			1 (3)		6	
	Cis bisexual man	6 (5.2)		8 (5.6)		14 (5.4)		3 (6)			2 (6.1)		5	
	Cis lesbian	2 (1.7)		1 (0.7)		3 (1.2)		2 (4)			0 (0)		2	
	Transgender heterosexual woman	1 (0.9)		0 (0)		1 (0.4)		0 (0)			0 (0)		0	
**Sex assigned at birth, n (%)**														
	Male	83 (71.6)		110 (77.5)		193 (74.8)		37 (74)			24 (72.7)		61	
	Female	33 (28.5)		32 (22.5)		65 (25.2)		13 (26)			9 (27.3)		22	
**Intersex, n (%)**														
	No	114 (98.3)		134 (94.4)		248 (96.1)		49 (98)			32 (97)		81	
	Prefer not to answer	2 (1.7)		3 (2.1)		5 (1.9)		1 (2)			0 (0)		1	
	Yes	0 (0)		5 (3.5)		5 (1.9)		0 (0)			1 (3)		1	
**Education, n (%)**														
	Did not complete high school	10 (8.6)		16 (11.3)		26 (10.1)		4 (8)			2 (6.1)		6	
	High school	26 (22.4)		41 (28.9)		67 (26)		13 (26)			9 (27.3)		22	
	Trade certificate or diploma	35 (30.2)		37 (26.1)		72 (27.9)		13 (26)			12 (36.4)		25	
	Undergraduate degree	27 (23.3)		25 (17.6)		52 (20.2)		11 (22)			7 (21.2)		18	
	Postgraduate degree	18 (15.5)		23 (16.2)		41 (15.9)		9 (18)			3 (9.1)		12	
**MA^b^ use, mean (SD)**														
	MA use in the past 28 days (days)	15.7 (8.8)		14.3 (8.9)		14.9 (8.9)		15.8 (8.9)			15.4 (8.2)		—^c^	
	Age of first MA use (years)	26.4 (9.3)		26.5 (10.6)		26.4 (10)		28.9 (9)			27.5 (10.9)		—	
**History of treatment for problems related to MA use, n (%)**														
	No	74 (63.3)		85 (59.9)		159 (61.6)		35 (68.6)			24 (72.7)		59	
	Yes	43 (36.8)		57 (40.1)		100 (38.8)		16 (31.4)			9 (27.3)		25	
**History of injecting MA in past 4 weeks, n (%)**														
	No	50 (42.7)		65 (45.8)		115 (44.6)		21 (41.2)			9 (27.3)		30	
	Yes	67 (57.3)		77 (54.2)		144 (55.8)		30 (58.8)			24 (72.7)		54	

^a^Baseline and day 28 demographic data on age, gender identity, sexuality, sex assigned at birth, intersex status, education, and days of methamphetamine use in the previous 28 days is missing for 1 participant.

^b^MA: methamphetamine.

^c^Not applicable.

### Primary Outcomes

The primary outcomes for help-seeking were assessed using the 84 participants retained to day 28 including 33 (39.2%) participants in the intervention group and 51 (60.7%) participants in the control group.

Table 3 compares actual help-seeking (mAHSQ), intention to seek help (mGHSQ), and motivation to change (mRR) between the 2 groups at baseline and day 28*.* A greater proportion of participants in the intervention group recently sought professional help (mAHSQ) at day 28 than in the control group (n=15, 45.5% vs n=12, 23.5%; *χ*^2^_1_=4.42; *P*=.04).

Regression analyses for mAHSQ, mGHSQ, and mRR are presented in Table 4. In the primary regression analyses, there was no statistically significant difference in the odds of actual help-seeking, intention to seek help, or motivation to change between the immediate access group and the waitlist control group on day 28.

**Table 3:** Help-seeking, intention to seek help, and motivation to change.

			Actual help-seeking (mAHSQ^a^)									Intention to seek help (mGHSQ^b^)^c^									Motivation to change (mRR^d^)^e^		
			Professional		*P* value		Nonprofessional		*P* value		Professional			*P* value		Nonprofessional		*P* value		Motivation to change			*P* value
**Baseline, n (%)**					.23				.22					.19				.43					≥.99
	Control	11 (21.6)				15 (29.4)				30 (58.8)					23 (45.1)				17 (33.3)				
	Intervention	11 (33.3)				14 (42.4)				24 (72.7)					23 (36.4)				11 (33.3)				
**Day 28, n (%)**					.04				.31					.45				.90					.09
	Control	12 (23.5)				22 (43.1)				32 (62.8)					27 (52.9)				14 (27.4)				
	Intervention	15 (45.5)				18 (54.6)				28 (54.6)					27 (51.5)				15 (45.4)				

^a^mAHSQ: modified Actual Help Seeking Questionnaire.

^b^mGHSQ: modified General Help Seeking Questionnaire.

^c^Modified General Help Seeking Questionnaire categorized as 1-4, no intention to seek help; 5-7, intention to seek help.

^d^mRR: modified readiness ruler.

^e^Modified readiness ruler categorized as <8, not motivated to change; ≥8, motivated to change.

**Table 4:** Logistic regression for mAHSQ^a^, mGHSQ^b^, and mRR^c^.

Outcome measure and outcome response set		Adjusted odds ratio^d^ (95% CI)	*P* value
**mAHSQ**			
	Professional help-seeking–actual	2.72 (0.86-8.64)	.09
	Nonprofessional help-seeking–actual	1.21 (0.44-3.67)	.71
**mGHSQ^e^**			
	Professional help-seeking–intention	0.49 (0.17-1.32)	.17
	Nonprofessional help-seeking–intention	1.04 (0.42-2.64)	.93
**mRR^f^**			
	Motivation to change	2.4 (0.89-6.47)	.08

^a^mAHSQ: modified Actual Help Seeking Questionnaire.

^b^mGHSQ: modified General Help Seeking Questionnaire.

^c^mRR: modified readiness ruler.

^d^Adjusted for baseline response.

^e^Score on a 7-point Likert scale categorized as 1-4, no intention to seek help; 5-7, intending to seek help.

^f^mRR scored as <8, not motivated to change; ≥8, motivated to change.

### Ancillary Analysis

Based on the multiple imputed data set, consisting of 10 baseline characteristics, an ancillary analysis was conducted. While overall the results were consistent with our observed case analysis, there was a significant difference in the odds of recent professional help-seeking between the immediate access group compared to the waitlist control group on this analysis (adjusted odds ratio [aOR] 2.64, 95% CI 1.19-5.83, *P*=.02; Table S2 in Multimedia Appendix 1).

### Secondary Outcomes

The most commonly accessed features of the app over this study’s period are presented in Table S3 in Multimedia Appendix 1. The most accessed features were the dashboard feature, information, and methamphetamine tracking resources.

There were no between-group changes in days of methamphetamine use at day 28, as presented in Table S4 in Multimedia Appendix 1.

In a multivariable regression analysis with app engagement time as the response variable, participants who identified as gay males had 68% more app engagement time than all other participants (ratio 1.68, 95% CI 1.04-2.98, *P*=.03). It was not possible to analyze S-Check app engagement by other sexualities and gender identities due to small numbers. Greater days of methamphetamine use at baseline was also found to be a predictor of S-Check app engagement, with 3% more app engagement time for each additional day of methamphetamine use (ratio 1.03, 95% CI 1.00-1.06, *P*=.03). Participants who intended to seek professional help (mGHSQ) at baseline had 17% more S-Check app engagement than those who did not (ratio 1.17, 95% CI 1.03-1.32, *P*=.01). After adjusting for baseline days of methamphetamine use, a 10-minute increase in app engagement time over the 28-day intervention was associated with a decrease in days of methamphetamine use by 0.4 days (regression coefficient [β] –0.04, *P*=.02; intervention group: n=33). In a subgroup analysis of participants who were not seeking help at baseline, for every additional minute of S-Check app use, there was an 8% increase in the likelihood of seeking professional help at day 28 (ratio 1.08, 95% CI 1.02-1.22, *P*=.04).

## Discussion

### Principal Findings

This study demonstrated that compared to waitlist controls, almost twice the proportion of participants with immediate access to the S-Check app sought professional help by day 28. Importantly, among participants not seeking help at baseline, each minute in the app was associated with seeking professional help by day 28, and there was an association between increased S-Check app use and decreased methamphetamine use over the 28 days among those in the immediate access group.

This study has limitations. We aimed to recruit 510 participants but we were only able to achieve a sample of 259. Retention to the primary end point was 32%, less than the expected 50%, which was likely unrealistic, given emerging evidence for this type of study suggests that when participants are enrolled in mHealth studies after downloading an app, retention is generally <30% [52]. Future research may include methods whereby participants are recruited before downloading the app; however, this design would affect the generalizability of the results to the population recruited. We learned important lessons about implementing an app-based trial. We underestimated the resources required to support a study of this nature. Keeping this study open longer may have increased our sample size. Budgeting for longer periods of recruitment and advertising may support higher enrollment in future trials. Apps to address substance use disorder should be co-designed with the community anticipated to benefit from their use. To this end, the stepped process of consultation with affected populations in transitioning from a clinical intervention to a smartphone app is a strength of the S-Check app. Further refinement of this resource or subsequent apps to address methamphetamine use should include active engagement with people who will use the app, to ensure alignment of what will benefit this population and what research and developers are delivering.

There is a possibility that this sample was already motivated toward change. Given the design of the app encourages people to understand more about their methamphetamine use and its impact, those who downloaded it likely had some interest in how their methamphetamine use was affecting them. In selecting a primary outcome, future studies of the S-Check app may therefore seek to examine how people use the app to better understand their methamphetamine use, identify which aspects of their use may be problematic for them, and how the app provides earlier insights into the relationships between methamphetamine use and aspects of their life. In the present study, assessing help-seeking and motivation to change is difficult given fixed time points and in binary measures as is demanded of a randomized controlled trial, while change is a fluid process. Engaging with the app may in itself have addressed concerns for participants, reducing the need or motivation to seek external help. Further exploration of the place of a self-administered information app such as the S-Check app within the broader treatment landscape in support of people who consume methamphetamine is warranted. A nuanced understanding of progress along the change continuum, an aim of the app, is required in future studies.

Sample characteristics limit the generalizability of the results to the broader Australian population of people who use methamphetamine. The majority of participants recruited to our study identified as male (69.9%) while 64% of past-month methamphetamine use occurs among men in Australia [53]. Our sample had a large proportion of participants who had undertaken higher level education (35.9% had a University degree), as compared to underlying population data (24%) [53]. In our study, neither ethnicity nor employment data were collected, therefore we cannot comment on the generalizability of these findings to a range of communities affected by methamphetamine use. This study recruited a large proportion of participants identifying as gay men (n=131, 50.8% of the sample), reflecting the recruitment strategy through targeted social media channels. The underrepresentation of women, transgender people, or other populations may be attributed to our recruitment strategy, but we do not have any way of knowing whether this is due to the design or content of the app. It is also possible that people did not want to use an app that would be associated with their drug use, particularly given the legal implications of drug use. Nevertheless, the anonymous nature of the S-Check app may reach participants who are reluctant to seek traditional or face-to-face treatment services due to barriers such as high cost, treatment access delays (eg, long waitlists), travel time to services, or fear of stigma.

The S-Check app recruited participants who had predominantly never sought treatment for methamphetamine use previously, and who reported injecting methamphetamine in the 4 weeks to baseline, a potential surrogate for severity of dependence. Furthermore, a higher proportion of those who were retained in the intervention group were people who injected methamphetamine. Previous studies have demonstrated greater methamphetamine-related service use among people who inject methamphetamine [54,55]. This may be due to the lack of treatment options for methamphetamine use disorders, whereas harm reduction services are more accessible, such as NSPs to reduce injection-related harms [56]. Our recruitment strategy included advertising through NSPs, and this may reflect that. However, our results suggest that the S-Check app can act as a feasible low-resource, early intervention for participants who may be reluctant to seek traditional or face-to-face services, or are not linked to services such as those by referral points that may be made available at NSPs.

While it was not designed to be a motivator for reducing methamphetamine use, participants who spent more time engaged with the S-Check app demonstrated a reduction in methamphetamine use from baseline to day 28. This finding is consistent with other mHealth apps that included self-monitoring for alcohol and other substance use [57], reinforcing the theory that supportive self-monitoring influences behavior change [58,59]. Importantly, participants who had more frequent methamphetamine use engaged for longer with the S-Check app than those with less frequent use; and those participants who reported injecting methamphetamine were more likely to be retained to day 28 if they were randomized to the intervention group than those who did not report injecting. These findings highlight a potential treatment gap that could be filled by an mHealth intervention such as the S-Check app, or at the very least the potential of the S-Check app to be an adjunct to other treatment and harm reduction services and an important and accessible resource for when those are not available. The S-Check app was intended as an early intervention but may be an attractive resource for those people who are more frequent or injecting consumers of methamphetamine as well.

Previous research has highlighted a need for targeted interventions to improve pathways to professional support among people who use methamphetamine [55]. While we collected baseline data on 259 participants, 560 people downloaded the app. Of those who completed screening, only 10 were ineligible. This suggests that the S-Check app was highly accessible, while perhaps recruitment into the research study was not favorable; for example, it is unknown if potential participants did not proceed due to the chance of being randomized to the waitlist control. While not statistically significant, a higher proportion of those randomized to the waitlist went on to complete the day 28 assessment. It cannot be inferred from our data whether this is related to satisfaction of those randomized to immediately access the app, or perhaps reflecting the context of the waitlist group wanting to access the app.

Future directions include exploring the incorporation of the S-Check app as part of a stepped-care approach to clinical treatment, within a range of clinical settings, or as a motivational and self-awareness tool to be provided to people waiting for care. Primary care and sexual health service use is high among people who use methamphetamine, but opportunities to intervene are often missed. A health economic analysis was not part of the scope of this project and as such more work is required to test and analyze the costing and implementation of the app. Further development of the S-Check app and dissemination more broadly as a freely accessible resource to support people in understanding their methamphetamine use is planned.

### Conclusions

This study demonstrates the effectiveness of the S-Check app in motivating help-seeking, particularly among treatment-naïve populations. The S-Check app was shown to be a feasible, low-resource, self-administered intervention for adults in Australia who use methamphetamine. Using supportive self-monitoring, the S-Check app assists people who use methamphetamine to identify problem use, shifting people along the motivational pathway, and promoting treatment seeking. As an early intervention, the S-Check app has the potential to facilitate the first step in a stepped-care approach to treatment, reducing treatment delays and avoiding more serious adverse health outcomes. Further work is required to demonstrate the generalizability of the S-Check app among people who use methamphetamine in Australia and to increase access to other internet-enabled devices.

## Appendix

Multimedia Appendix 1 CONSORT (Consolidated Standards of Reporting Trials) checklist and tables.

Multimedia Appendix 2 CONSORT eHEALTH checklist V 1.6.1.

## Abbreviations

CONSORT: Consolidated Standards of Reporting Trials
ITT: intention-to-treat
mAHSQ: modified Actual Help Seeking Questionnaire
mGHSQ: modified General Help Seeking Questionnaire
mRR: modified readiness ruler
NSP: needle and syringe program
