# Supplementary material for: Effect of a Smartphone App (S-Check) on Actual and Intended Help-Seeking and Motivation to Change Methamphetamine Use Among Adult Consumers of Methamphetamine in Australia: Randomized Waitlist-Controlled Trial
Source: JMIR Mhealth Uhealth. 2024 Jul 3;12:e55663. doi: 10.2196/55663 (PMC11255525; doi:10.2196/55663)
Supplement: Multimedia Appendix 1 [file mhealth_v12i1e55663_app1.docx]

**Table S1.** CONSORT checklist. CONSORT: Consolidated Standards of Reporting Trials.

| Section/Topic | Item No | Checklist item | Reported on page No |
| --- | --- | --- | --- |
| **Title and abstract** | | | |
|  | 1a | Identification as a randomised trial in the title | 1 |
|  | 1b | Structured summary of trial design, methods, results, and conclusions (for specific guidance see CONSORT for abstracts) | 3-4 |
| **Introduction** | | | |
| Background and objectives | 2a | Scientific background and explanation of rationale | 5-6 |
|  | 2b | Specific objectives or hypotheses | 6-7 |
| **Methods** | | | |
| Trial design | 3a | Description of trial design (such as parallel, factorial) including allocation ratio | 7 |
|  | 3b | Important changes to methods after trial commencement (such as eligibility criteria), with reasons | NA |
| Participants | 4a | Eligibility criteria for participants | 7 |
|  | 4b | Settings and locations where the data were collected | 7-8 |
| Interventions | 5 | The interventions for each group with sufficient details to allow replication, including how and when they were actually administered | 8; Table 1; Figures 1-3 |
| Outcomes | 6a | Completely defined pre-specified primary and secondary outcome measures, including how and when they were assessed | 8-10 |
|  | 6b | Any changes to trial outcomes after the trial commenced, with reasons | NA |
| Sample size | 7a | How sample size was determined | 9 |
|  | 7b | When applicable, explanation of any interim analyses and stopping guidelines | NA |
| Randomisation: |  |  |  |
| Sequence generation | 8a | Method used to generate the random allocation sequence | 10 |
|  | 8b | Type of randomisation; details of any restriction (such as blocking and block size) | 10 |
| Allocation concealment mechanism | 9 | Mechanism used to implement the random allocation sequence (such as sequentially numbered containers), describing any steps taken to conceal the sequence until interventions were assigned | 10 |
| Implementation | 10 | Who generated the random allocation sequence, who enrolled participants, and who assigned participants to interventions | 10 |
| Blinding | 11a | If done, who was blinded after assignment to interventions (for example, participants, care providers, those assessing outcomes) and how | NA |
|  | 11b | If relevant, description of the similarity of interventions | NA |
| Statistical methods | 12a | Statistical methods used to compare groups for primary and secondary outcomes | 10-11 |
|  | 12b | Methods for additional analyses, such as subgroup analyses and adjusted analyses | 11 |
| **Results** | | | |
| Participant flow (a diagram is strongly recommended) | 13a | For each group, the numbers of participants who were randomly assigned, received intended treatment, and were analysed for the primary outcome | Figure 4 |
|  | 13b | For each group, losses and exclusions after randomisation, together with reasons | Figure 4 |
| Recruitment | 14a | Dates defining the periods of recruitment and follow-up | - |
|  | 14b | Why the trial ended or was stopped | NA |
| Baseline data | 15 | A table showing baseline demographic and clinical characteristics for each group | Table 2 |
| Numbers analysed | 16 | For each group, number of participants (denominator) included in each analysis and whether the analysis was by original assigned groups | 11 |
| Outcomes and estimation | 17a | For each primary and secondary outcome, results for each group, and the estimated effect size and its precision (such as 95% confidence interval) | 12 |
|  | 17b | For binary outcomes, presentation of both absolute and relative effect sizes is recommended | - |
| Ancillary analyses | 18 | Results of any other analyses performed, including subgroup analyses and adjusted analyses, distinguishing pre-specified from exploratory | 12 |
| Harms | 19 | All important harms or unintended effects in each group (for specific guidance see CONSORT for harms) | NA |
| **Discussion** | | | |
| Limitations | 20 | Trial limitations, addressing sources of potential bias, imprecision, and, if relevant, multiplicity of analyses | 13-14 |
| Generalisability | 21 | Generalisability (external validity, applicability) of the trial findings | 14 |
| Interpretation | 22 | Interpretation consistent with results, balancing benefits and harms, and considering other relevant evidence | 15-16 |
| **Other information** | | |  |
| Registration | 23 | Registration number and name of trial registry | 4, 7 |
| Protocol | 24 | Where the full trial protocol can be accessed, if available | NA |
| Funding | 25 | Sources of funding and other support (such as supply of drugs), role of funders | 17 |

**Table S2.** 28-day effectiveness of the S-Check app to motivate professional help-seeking, intention to seek help, readiness to change, and change in days of methamphetamine use based on multiple imputed data sets.

| Intervention vs. Control group (n=259) | Regression coefficient β | Odds Ratio | 95% CI^a^ | *P-*value |
| --- | --- | --- | --- | --- |
| Professional help-seeking | 0.97 | 2.64 | 1.19, 5.83 | **.017** |
| Non-professional help-seeking | 0.29 | 1.33 | 0.69, 2.55 | .39 |
| Intention to seek professional help | -0.33 | - | -1.34, 0.68 | .51 |
| Intention to seek non-professional help | 0.07 | - | -0.80, 0.94 | .87 |
| Motivation to change | -0.10 | - | -1.63, 1.43 | .89 |
| Change in days of methamphetamine use | 0.27 | - | -4.95, 5.49 | .92 |

^a^ 95% CI, 95% Confidence Interval

**Table S3.** Frequency of use of nonresearch related S-Check app features.

|  | | Frequency of times viewed^a^ | Percentage of total frequency (%) |
| --- | --- | --- | --- |
| Dashboard | | **4500** | **29.11** |
| Resources | | **3463** | **22.40** |
|  | Meth use | 785 | 30.66 |
|  | Psychological wellbeing | 577 | 22.54 |
|  | Sexual health | 370 | 14.45 |
|  | My thinking | 320 | 12.50 |
|  | Social health | 306 | 11.95 |
|  | Physical health | 202 | 7.89 |
| Layers | | **3455** | **22.35** |
|  | MethMethodology | 493 | 14.27 |
|  | Let's begin | 336 | 9.73 |
|  | Reality Check | 303 | 8.77 |
|  | Sexual Behaviour | 228 | 6.60 |
|  | Your Body | 228 | 6.60 |
|  | Day to Day Activities | 175 | 5.07 |
|  | Cognitive Health | 151 | 4.37 |
|  | Overview | 146 | 4.23 |
|  | How I Feel About My Use | 144 | 4.17 |
|  | Stress & Anxiety | 140 | 4.05 |
|  | Sex & Drugs | 134 | 3.88 |
|  | My Mood Rating | 118 | 3.42 |
|  | How you take your [meth] | 115 | 3.33 |
|  | Your medical history | 111 | 3.21 |
|  | My Use | 109 | 3.15 |
|  | Am I Feeling OK? | 108 | 3.13 |
|  | My General Wellbeing | 108 | 3.13 |
|  | Your family's health | 108 | 3.13 |
|  | Overall Satisfaction | 102 | 2.95 |
|  | My Mental Health | *98* | *2.84* |
| Journal | | 1116 | 7.22 |
| Reports | | 688 | 4.45 |
| Check-in | | 662 | 4.28 |
| Settings | | 521 | 3.37 |
| Help | | 430 | 2.78 |
| Referral | | 424 | 2.74 |
| Tutorial | | 198 | 1.28 |

^a^ Both intervention and control groups, within the 56-day study period

**Table S4.** Between group differences in changes of methamphetamine use at day 28.

| Time point | Study Group | N^a^ | Days of methamphetamine use | Days of methamphetamine use | *P-*value |
| --- | --- | --- | --- | --- | --- |
|  |  |  | Mean (SD) | Median |  |
| Baseline | Control | 116 | 15.7 (8.8) | 16 |  |
|  | Intervention | 142 | 14.3 (9.0) | 15 | .22 |
| Baseline^b^ | Control | 51 | 15.8 (8.9) | 16 |  |
|  | Intervention | 33 | 15.4 (8.2) | 15 | .83 |
| 28 Days | Control | 51 | 15.3 (9.2) | 15 |  |
|  | Intervention | 33 | 15.8 (8.2) | 16 | .78 |

^a^ model adjusted for baseline days of methamphetamine use

^b^ baseline data for participants retained to 28 days
